# Effects of intraoperative lung-protective ventilation on clinical outcomes in patients with traumatic brain injury: a randomized controlled trial

**DOI:** 10.1186/s12871-021-01402-w

**Published:** 2021-06-28

**Authors:** Lulu Jiang, Yujuan Wu, Yang Zhang, Dahao Lu, Keshi Yan, Ju Gao

**Affiliations:** 1 Department of Anesthesiology, the Second Xiangya Hospital, Central South University, 139# Renmin Central Road, 410011 Changsha, China; 2 Department of Anesthesiology, Northern Jiangsu People’s Hospital, Clinical Medical School, Yangzhou University, 98# Nantong West Road, 225001 Yangzhou, China; 3 Department of Anesthesiology, Xiangtan Central Hospital, 120# Heping Road, 411100 Xiangtan, China

**Keywords:** Traumatic brain injury, Lung-protective ventilation, Postoperative pulmonary complications, Optic nerve sheath diameter, Glial fibrillary acidic protein, Ubiquitin carboxyl-terminal hydrolase isozyme L1

## Abstract

**Background:**

Secondary lung injury is the most common non-neurological complication after traumatic brain injury (TBI). Lung-protective ventilation (LPV) has been proven to improve perioperative oxygenation and lung compliance in some critical patients. This study aimed to investigate whether intraoperative LPV could improve respiratory function and prevent postoperative complications in emergency TBI patients.

**Methods:**

Ninety TBI patients were randomly allocated to three groups (1:1:1): Group A, conventional mechanical ventilation [tidal volume (VT) 10 mL/kg only]; Group B, small VT (8 mL/kg) + positive end-expiratory pressure (PEEP) (5 cmH_2_O); and Group C, small VT (8 mL/kg) + PEEP (5 cmH_2_O) + recruitment maneuvers (RMs). The primary outcome was the incidence of total postoperative pulmonary complications; Secondary outcomes were intraoperative respiratory mechanics parameters and serum levels of brain injury markers, and the incidence of each postoperative pulmonary and neurological complication.

**Results:**

Seventy-nine patients completed the final analysis. The intraoperative PaO_2_ and dynamic pulmonary compliance of Groups B and C were higher than those of Group A (*P* = 0.028; *P* = 0.005), while their airway peak pressure and plateau pressure were lower than those of group A (*P* = 0.004; *P* = 0.005). Compared to Group A, Groups B and C had decreased 30-day postoperative incidences of total pulmonary complications, hypoxemia, pulmonary infection, and atelectasis (84.0 % vs. 57.1 % vs. 53.8 %, *P* = 0.047; 52.0 % vs. 14.3 % vs. 19.2 %, *P* = 0.005; 84.0 % vs. 50.0 % vs. 42.3 %, *P* = 0.006; 24.0 % vs. 3.6 % vs. 0.0 %, *P* = 0.004). Moreover, intraoperative hypotension was more frequent in Group C than in Groups A and B (*P* = 0.007). At the end of surgery, the serum levels of glial fibrillary acidic protein and ubiquitin carboxyl-terminal hydrolase isozyme L1 in Group B were lower than those in Groups A and C (*P* = 0.002; *P* < 0.001). The postoperative incidences of neurological complications among the three groups were comparable.

**Conclusions:**

Continuous intraoperative administration of small VT + PEEP is beneficial to TBI patients. Additional RMs can be performed with caution to prevent disturbances in the stability of cerebral hemodynamics.

**Trial registration:**

Chinese Clinical Trial Registry (ChiCTR2000038314), retrospectively registered on September 17, 2020.

## Background

Traumatic brain injury (TBI) is a major medical and socioeconomic problem. Over 50 million people worldwide experience TBI every year, and the morbidity has increased in the past decade [1]. TBI causes a wide range of systemic effects. It was reported that 89 % of severe TBI patients experienced at least one non-neurological complication, of which 81 % developed respiratory dysfunction, including 23 % of respiratory failure cases. Hence, respiratory complications are prevalent non-neurological disorders experienced after TBI [2]. In addition, neural and humoral regulation after injury leads to an attenuated response of lung tissues to stress [3, 4], thus increasing the risk of pulmonary complications, especially pulmonary infection, neurogenic pulmonary edema (NPE), ventilator-associated lung injury (VALI), and atelectasis.

In general anesthesia, tidal volume (VT) was usually set at 10-15mL/kg corrected body weight (CBW) before, which is higher than that of most mammals with spontaneous respiration. High VT ventilation may cause alveolar overdistention, inflammatory mediator spillover, and VALI. Currently, lung-protective ventilation (LPV) is defined as VT ≤ 8mL/kg, positive end-expiratory pressure (PEEP) ≥ 5cmH_2_O, and airway plateau pressure (Pplat) ≤ 30cmH_2_O, which is recognized as the optimal ventilation mode for patients with acute respiratory distress syndrome (ARDS) in the intensive care unit (ICU) [5]. In view of satisfactory application of LPV in ARDS patients, perioperative lung protection in the operating room has also been highlighted by anesthesiologists.

Various factors contribute to the complex interactions between mechanical ventilation (MV) and cerebral hemodynamics during surgery. Many clinical trials or meta-analyses have found that perioperative application of LPV can improve intraoperative oxygenation and lung compliance, and relieve postoperative pulmonary complications (PPCs). However, PEEP or recruitment maneuvers (RMs) may disrupt the stability of cerebral hemodynamics in emergency TBI patients, who are often excluded from these studies. Whether perioperative LPV is also beneficial to these patients requires explorations. A recent retrospective study involving 28,644 TBI patients in the ICU showed no significant changes in intracranial pressure (ICP) and cerebral perfusion pressure (CPP) after applying LPV, indicating the safety of respiratory support in TBI patients [6].

Here, we conducted a randomized controlled trial with the hypothesis that intraoperative use of LPV can improve respiratory function and prevent postoperative complications in TBI patients. The primary aim was to assess the incidence of total PPCs in TBI patients treated with LPV. The secondary aims were to investigate intraoperative respiratory mechanics parameters and serum levels of brain injury markers, and the incidence of each postoperative pulmonary and neurological complication.

## Methods

### Study design, approvals and registration

A single-center, randomized controlled study involving 90 TBI patients was approved by the Ethics Committee of Northern Jiangsu People’s Hospital (2,019,113). Informed consent was obtained from patients or their relatives. The trial was retrospectively registered at the Chinese Clinical Trial Registry (ChiCTR2000038314) on 17/09/2020.

### Patients

TBI patients aged 18–65 years who underwent emergency intracranial evacuation of hematoma were enrolled. No limitation on sex was set. Their body mass index (BMI) ranged from 18.5 to 29.9 kg/m^2^, and they were American Society of Anesthesiologists (ASA) Classification III or IV.

Patients with a history of mental diseases or other neurological disorders (epilepsy, dementia, cerebrovascular malformation, etc.), severe cardiovascular diseases (valvular heart disease, pericarditis, cor pulmonale, etc.), and severe hepatic or renal insufficiency (cirrhosis, chronic renal failure, nephrotic syndrome, etc.), were excluded. Patients who had stroke, myocardial infarction or major surgery within three months and refused to participate were also excluded.

Patients were assigned by computer-generated randomized sequence to three groups (1:1:1): Group A (conventional MV), Group B (small VT + 5 cmH_2_O PEEP), and Group C (small VT + 5 cmH_2_O PEEP + RMs). The random allocation scheme was sealed by the principal investigator in opaque envelopes. Two experienced anesthesiologists blinded to the random allocation enrolled eligible participants, and the other two assigned participants to interventions.

### Anesthesia

Midazolam (0.05 mg/kg), sufentanil (0.5 µg/kg), propofol (1–2 mg/kg), and cisatracurium (0.15–0.20 mg/kg) were intravenously injected for anesthesia induction. After tracheal intubation, the anesthesia machine was connected to start MV.

Inhalation of 2 % sevoflurane during operation, and intravenous pump injection of remifentanil (0.1–0.3 µg/kg/min), dexmedetomidine (0.01 µg/kg/min), and cisatracurium (5 µg/kg/min) were performed for anesthesia maintenance, the dose of which was adjusted according to the depth of anesthesia. Vasoactive drugs were applied if cyclic fluctuations occurred during surgery.

### Mechanical ventilation

All patients received MV after tracheal intubation under the same general anesthesia management. Immediately after intubation, volume-controlled ventilation was applied in three groups with VT 10 mL/kg CBW, inspiration/expiration 1:2, fraction of inspired oxygen 100 %, oxygen flow 2 L/min, and no PEEP or RMs. Five minutes later, the parameters of Groups B and C were continuously adjusted to 8 mL/kg CBW of VT and 5 cmH_2_O PEEP. Patients in Group C received two RMs before opening and after closing the endocranium. Briefly, RMs were performed to maintain an airway pressure of 30 cmH_2_O for 30 s. During the operation, the respiratory rate was adjusted according to arterial blood gas analysis to maintain end-tidal carbon dioxide partial pressure (P_ET_CO_2_) at 30–35 mmHg.

Postoperative removal of the tracheal catheter was discussed by the anesthesiologist and the attending physician. When patients recovered spontaneous breathing, swallowing, cough reflex, VT > 6 mL/kg, oxygen saturation (SpO_2_) > 95 % for 10 min, gentle suction to clean the tube and oropharyngeal secretions, removal of the tracheal catheter, and delivery of oxygen by mask at 5 L/min were performed. The mask was removed 10 min later, followed by 20 min of observation, and then patients were sent to the ward. Others with tracheal catheter were directly sent to the ICU in the case of sedation, analgesia and ventilator.

### Outcomes

The primary outcome was the incidence of 30-day total PPCs, which included hypoxemia, pulmonary infection, atelectasis, ARDS, VALI, and NPE. The definition of each PPC was as follows. (1) Hypoxemia: arterial partial pressure of oxygen (PaO_2_) < 60 mmHg or SpO_2_ < 90 % in room air [7]. (2) Pulmonary infection: the clinical pulmonary infection score was greater than 6, and the symptoms of infection started before 48 h of respiratory treatment. (3) Atelectasis: the chest X-ray or computed tomography images showed lung opacification with a shift of the mediastinum, hilum or hemidiaphragm toward the affected area, and compensatory overinflation in the adjacent non-atelectatic lung. (4) ARDS: acute respiratory failure; PaO_2_/fraction of inspired oxygen ≤ 300 mmHg; bilateral infiltrates on chest X-ray, and no signs of heart failure [8]. (5) VALI: mechanical ventilation > 48 h; pulmonary interstitial emphysema, pneumomediastinum, subcutaneous emphysema or pneumothorax, and infiltrates on chest X-ray. (6) NPE: the symptoms included dyspnea, tachypnea, cyanosis, and rales, crackles, or rhonchi. PaO_2_/partial pressure of inspired oxygen < 200; mild leukocytosis; bilateral alveolar opacities and diffuse alveolar infiltrates without cardiomegaly on chest X-ray [9].

Secondary outcomes were (1) intraoperative oxygenation and respiratory mechanics parameters [PaO_2_, arterial partial pressure of carbon dioxide (PaCO_2_), pulmonary dynamic compliance (Cdyn), airway peak pressure (Ppeak), Pplat, heart rate, mean arterial pressure (MAP)]; (2) the incidences of intraoperative pulmonary and cardiovascular adverse reactions [SpO_2_ < 90 % or P_ET_CO_2_ > 45 mmHg or systolic blood pressure (SBP) < 90 mmHg for more than 1 min, any arrhythmia]; (3) intraoperative serum levels of brain injury markers [glial fibrillary acidic protein (GFAP), ubiquitin carboxyl-terminal hydrolase isozyme L1 (UCHL1)]; and (4) the 30-day postoperative incidences of pulmonary infection, hypoxemia, atelectasis, ARDS, VALI, NPE, intracranial infection, intracranial hypertension, epilepsy, encephaledema, and reoperation.

Other outcomes included intraoperative optic nerve sheath diameter (ONSD), postoperative duration of MV, length of stay, 30-day Glasgow Outcome Scale Extended (GOSE).

### Data collection

The baseline characteristics were sex, age, BMI, ASA class, preoperative Glasgow Coma Scale score, hemoglobin concentration, intraoperative bleeding volume and infusion quantity, and total operative and anesthesia time.

Blood gas analysis was performed on a 1 mL radial artery blood sample at the onset of MV (T_1_), ventilation for 60 min (T_2_), and the end of surgery (T_3_). The ONSD was measured by color Doppler ultrasound at anesthesia induction (T_0_), T_1_, after applying PEEP (t_0_), before the first RM (t_1_), after the first RM (t_2_), before the second RM (t_3_), after the second RM (t_4_) and T_3_. Five milliliters internal jugular vein blood sample of each patient at T_1_, T_2_ and T_3_ was placed in vacuum blood collection tubes, and the supernatant was collected and detected by ELISA Kit of GFAP and UCHL1 (ab223867, Abcam; CY-8092, CircuLex) according to the corresponding instructions.

### Statistical analysis

Sample size calculation was based on the previous report [10] and our pilot trial, which showed the incidence of total PPCs among the three groups was 86.7 %, 53.3 and 40.0 %, respectively. According to the calculation formula for the comparison of multiple sample rates ($n=\frac{1641.4{\uplambda }}{{\left({\text{sin}}^{-1}\sqrt{{P}_{max}}-{\text{sin}}^{-1}\sqrt{{P}_{min}}\right)}^{2}}$) (λ = 12.65) [11], 23 patients per group were needed to detect a significant change in the incidence of total PPCs after applying LPV, with a type I error of 0.05 and 90 % power.

Statistical Package for the Social Sciences version 22.0 was used for data processing. Normally distributed measurement data are expressed as the mean ± standard deviation, and the Levene test was conducted to assess homogeneity. If the data met the hypothesis of equal variance, Student-Newman-Keuls was applied to compare differences between any two samples; otherwise, after performing the Kruskal-Wallis H test, the Bonferroni method was utilized to correct the significance level for post-hoc multiple comparisons. Measurement data with skew distributions are expressed as medians and interquartile ranges, which were compared in the same way as data with unequal variances. Enumeration data expressed as percentages were analyzed by the Chi-square test for R×C table data. Pairwise comparisons were conducted if all theoretical frequencies were greater than 5; otherwise, Fisher’s exact probability test and pairwise comparisons were conducted. *P* < 0.05 was considered statistically significant.

## Results

**Fig. 1:**
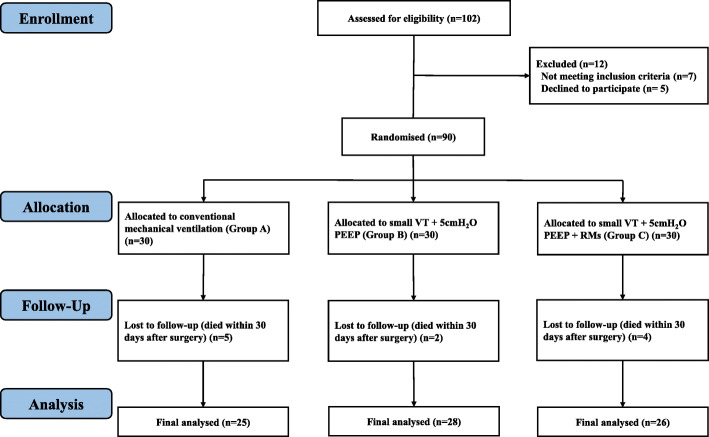
Consolidated Standards of Reporting Trials (CONSORT) flow diagram. PEEP, positive end-expiratory pressure; RMs, recruitment maneuvers; VT, tidal volume

From December 2019 to September 2020, we recruited 90 eligible TBI patients and assigned them equally to receive conventional MV (Group A), small VT + 5 cmH_2_O PEEP (Group B), and small VT + 5 cmH_2_O PEEP + RMs (Group C). Finally, 79 participants completed the final analysis, as 5, 2 and 4 patients died within 30 days postoperatively in Groups A, B and C, respectively (Fig. 1). The baseline characteristics of the participants were comparable (Table 1).

**Table 1:** Baseline characteristics by randomized group

	Group A (*n* = 25)	Group B (*n* = 28)	Group C (*n* = 26)	*P*
Sex, n (%)				
Male	17 (68.0)	20 (71.4)	20 (76.9)	0.772
Female	8 (32.0)	8 (28.6)	6 (23.1)	
Age, years, median (IQR)	55.0 (45.0–60.0)	52.5 (45.3–56.0)	50.0 (43.5–56.0)	0.500
BMI, kg/m^2^, mean ± SD	23.3 ± 2.1	22.9 ± 1.8	22.6 ± 1.8	0.469
ASA Class, n (%)				
III	9 (36.0)	7 (25.0)	7 (26.9)	0.649
IV	16 (64.0)	21 (75.0)	19 (73.1)	
Glasgow Coma Scale, n (%)^a^				
13–15	1 (4.0)	3 (10.7)	3 (11.5)	0.911
9–12	9 (36.0)	9 (32.1)	8 (30.8)	
≤8	15 (60.0)	16 (57.1)	15 (57.7)	
Preoperative hemoglobin, g/dL, median (IQR)	12.0 (10.0–13.0)	13.0 (11.3–13.8)	12.0 (11.0-13.3)	0.462
Intraoperative amount of bleeding, mL, median (IQR)	300.0 (200.0-400.0)	300.0 (200.0-437.5)	300.0 (200.0-500.0)	0.440
Intraoperative fluid infusion volume, mL, median (IQR)	2500.0 (1975.0-3175.0)	2500.0 (2000.0-3000.0)	2500.0 (2000.0-3000.0)	0.810
Operative time, min, median (IQR)	200.0 (150.0-237.5)	177.5 (156.3-199.5)	162.5 (150.0-222.5)	0.379
Anesthesia time, min, median (IQR)	245.0 (200.0-285.0)	220.0 (201.3-253.8)	202.5 (193.8–270.0)	0.508

*ASA* American Society of Anesthesiologists; *BMI* body mass index; *IQR* interquartile range; *SD* standard deviation

^a^ Glasgow Coma Scale score is an indicator used to assess the coma of a patient. It ranges from 3 to 15, and the higher the score, the better the consciousness. Scores of 13–15, 9–12 and ≤ 8 indicate mild, moderate and severe traumatic brain injury, respectively

**Fig. 2:**
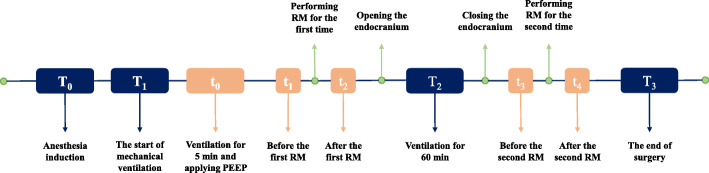
Timeline of intraoperative lung-protective ventilation strategy implementation. PEEP, positive end-expiratory pressure; RM, recruitment maneuver

Figure 2 shows the timeline of the intraoperative LPV strategy. Table 2 summarizes the intraoperative blood gas analysis, respiratory mechanics and hemodynamics. At T_1_, no significant differences in PaO_2_, PaCO_2_, Cdyn, Ppeak or Pplat were detected among the three groups. At T_2_, compared to Group A, the median PaO_2_ and Cdyn increased significantly in Groups B and C (336.0 vs. 375.5 vs. 388.0 mmHg, *P* = 0.028; 320.0 vs. 360.0 vs. 350.0 mL/cmH_2_O, *P* = 0.005), while their median Ppeak and Pplat decreased significantly (18.0 vs. 17.0 vs. 17.0 cmH_2_O, *P* = 0.004; 14.0 vs. 13.0 vs. 13.0 cmH_2_O, *P* = 0.005). No significant difference in PaCO_2_ was detected. At T_3_, the median PaO_2_, PaCO_2_, and Cdyn in Groups B and C were higher than those in Group A (340.0 vs. 397.5 vs. 402.5 mmHg, *P* = 0.005; 40.0 vs. 44.0 vs. 42.0 mmHg, *P* = 0.025; 330.0 vs. 340.0 vs. 340.0 mL/cmH_2_O, *P* = 0.009), which was opposite to the median Ppeak and Pplat (19.0 vs. 17.0 vs. 17.0 cmH_2_O, *P* = 0.012; 16.0 vs. 13.0 vs. 14.0 cmH_2_O, *P* = 0.003). There were no significant differences in the aforementioned indicators between Groups B and C at either T_2_ or T_3_. Meanwhile, heart rate and MAP among the three groups were comparable throughout the surgery.

**Table 2:** Intraoperative blood gas analysis, respiratory mechanics and hemodynamics

		Group A (*n* = 25)	Group B (*n* = 28)	Group C (*n* = 26)	*P*
PaO_2_, mmHg	T_1_	419.0 (381.5–486.0)	426.0 (400.8-450.3)	433.5 (367.5-487.3)	0.781
	T_2_	436.0 (382.0-487.0)	475.5 (447.3-496.8)^*^	488.0 (409.8-527.3)^*^	0.028
	T_3_	440.0 (394.5-493.5)	497.5 (486.8-534.5)^#^	502.5 (441.3–554.0)^*^	0.005
PaCO_2_, mmHg	T_1_	45.0 (39.5–50.0)	46.0 (40.3–49.0)	46.0 (37.0–49.0)	0.881
	T_2_	42.0 (39.0–46.0)	42.0 (40.0-47.8)	42.0 (38.8–47.3)	0.970
	T_3_	40.0 (38.0–42.0)	44.0 (39.3–47.8)^*^	42.0 (39.0-46.3)^*^	0.025
Cdyn, mL/cmH_2_O	T_1_	320.0 (300.0-335.0)	320.0 (310.0-350.0)	310.0 (300.0-322.5)	0.080
	T_2_	320.0 (295.0-355.0)	360.0 (332.5–370.0)^#^	350.0 (340.0-360.0)^*^	0.005
	T_3_	330.0 (305.0-345.0)	340.0 (330.0-360.0)^*^	340.0 (330.0-370.0)^*^	0.009
Ppeak, cmH_2_O	T_1_	17.0 (16.0–20.0)	18.0 (16.0–19.0)	19.0 (17.0–20.0)	0.379
	T_2_	18.0 (17.5–21.0)	17.0 (16.0-18.8)^#^	17.0 (15.8–19.0)^*^	0.004
	T_3_	19.0 (18.0–21.0)	17.0 (15.3–20.0)^*^	17.0 (16.0–19.0)^*^	0.012
Pplat, cmH_2_O	T_1_	13.0 (11.5–14.5)	14.0 (12.0-15.8)	15.0 (13.0-16.3)	0.068
	T_2_	14.0 (13.0–17.0)	13.0 (11.3–14.8)^*^	13.0 (11.0–15.0)^*^	0.005
	T_3_	16.0 (13.5–17.0)	13.0 (11.0–15.0)^#^	14.0 (11.8–15.3)^*^	0.003
Heart rate, min^− 1^	T_1_	81 (65–102)	80 (65–93)	76 (72–92)	0.779
	T_2_	66 (58–92)	70 (62–87)	68 (62–83)	0.891
	T_3_	64 (56–93)	65 (59–80)	68 (63–85)	0.421
MAP, mmHg^a^	T_1_	88.0 (77.0-101.5)	93.0 (85.0-101.8)	93.5 (88.5–109.0)	0.419
	T_2_	79.0 (73.5–90.0)	86.0 (77.0–95.0)	82.0 (73.8–86.5)	0.210
	T_3_	77.0 (71.0-92.5)	78.0 (71.3–84.3)	81.0 (72.0-91.5)	0.802

Data are presented as the median (interquartile range)

*Cdyn* pulmonary dynamic compliance; *DBP* diastolic blood pressure; *MAP* mean arterial pressure; *PaCO*_2_ arterial partial pressure of carbon dioxide; *PaO*_2_ arterial partial pressure of oxygen; *Ppeak* airway peak pressure; *Pplat* airway plateau pressure; *SBP* systolic blood pressure

^a^ MAP=(SBP + DBP*2)/3

^*^P < 0.05, ^#^*P* < 0.01 compared to Group A at the same point in time

Furthermore, intraoperative respiratory and cardiovascular adverse reactions were recorded and a 30-day postoperative follow-up was conducted (Table 3). Compared with that in Groups A and B, the incidence of intraoperative hypotension (SBP < 90 mmHg) in Group C increased significantly (32.0 % vs. 39.3 % vs. 73.1 %, *P* = 0.007), while no significant differences in the incidences of arrhythmia, SpO_2_ < 90 % and P_ET_CO_2_ > 45 mmHg were found among the three groups. Our follow-up results showed that the incidences of total PPCs, hypoxemia, pulmonary infection and atelectasis in Groups B and C were significantly lower than those in Group A (84.0 % vs. 57.1 % vs. 53.8 %, *P* = 0.047; 52.0 % vs. 14.3 % vs. 19.2 %, *P* = 0.005; 84.0 % vs. 50.0 % vs. 42.3 %, *P* = 0.006; 24.0 % vs. 3.6 % vs. 0.0 %, *P* = 0.004). However, the incidences of ARDS, VALI and NPE among the three groups were comparable. In addition, there were no significant differences in PPCs between Groups B and C. The postoperative incidences of neurological complications of the three groups were similar. The median postoperative ventilation time in Group A was significantly longer than that of Groups B and C (72.0 vs. 24.0 vs. 24.0 h, *P* = 0.006), which was comparable between the latter two groups. Likewise, there were no significant differences in GOSE score and hospital stay among the three groups.

**Table 3:** Intraoperative adverse reactions and 30-day postoperative follow-up

	Group A (*n* = 25)	Group B (*n* = 28)	Group C (*n* = 26)	*P*
Intraoperative adverse reactions, n (%)				
SpO_2_ < 90 %	2 (8.0)	1 (3.6)	1 (3.8)	0.685
P_ET_CO_2_>45 mmHg	1 (4.0)	5 (17.9)	5 (19.2)	0.213
SBP < 90mmHg	8 (32.0)	11 (39.3)	19 (73.1)^ab^	0.007
Arrhythmia	2 (8.0)	5 (17.9)	4 (15.4)	0.609
Postoperative pulmonary complications, n (%)				
Total	21 (84.0)	16 (57.1)^a^	14 (53.8)^a^	0.047
Hypoxemia	13 (52.0)	4 (14.3)^a^	5 (19.2)^a^	0.005
Pulmonary infection	21 (84.0)	14 (50.0)^a^	11 (42.3)^a^	0.006
Atelectasis	6 (24.0)	1 (3.6)^a^	0 (0.0)^a^	0.004
Acute respiratory distress syndrome	1 (4.0)	1 (3.6)	0 (0.0)	0.764
Ventilator-associated lung injury	3 (12.0)	3 (10.7)	2 (7.7)	0.902
Neurogenic pulmonary edema	1 (4.0)	0 (0.0)	1 (3.9)	0.537
Postoperative neurological complications, n (%)				
Intracranial infection	3 (12.0)	3 (10.7)	4 (15.4)	0.915
Intracranial hypertension	6 (24.0)	7 (25.0)	7 (26.9)	1.000
Epilepsy	2 (8.0)	2 (7.1)	3 (11.5)	0.890
Encephaledema	5 (20.0)	5 (17.9)	6 (23.1)	0.939
Reoperation	2 (8.0)	1 (3.6)	2 (7.7)	0.733
Other				
Mechanical ventilation time, h, median (interquartile range)	72.0 (36.0-105.0)	24.0 (3.0–62.0)^a^	24.0 (9.1–66.0)^a^	0.006
Length of stay, days, mean ± SD	21.5 ± 10.4	21.9 ± 8.3	22.0 ± 7.5	0.975
GOSE score, mean ± SD^c^	5 ± 1.6	6 ± 1.3	5 ± 1.2	0.768

*GOSE* Glasgow Outcome Scale Extended; *P*_*ET*_*CO*_*2*_ end-tidal carbon dioxide partial pressure; *SBP* systolic blood pressure; *SD* standard deviation; *SpO*_*2*_ oxygen saturation

^a^*P*<0.05 compared to Group A; ^b^*P*<0.05 compared to Group B

^c^ GOSE is used to assess outcomes of patients with brain damage and is divided into 8 levels. The higher the grade, the better the patient’s prognosis is

For ONSD (Table 4), there were no significant differences among the three groups at T_0_, T_1_ and T_3_. When compared within each group, the differences in Group A or B were comparable among time points. Comparisons in Group C were interesting. Specifically, after performing each RM, the mean ONSD (mm) at t_2_ or t_4_ was not only significantly higher than that before carrying out each RM (t_1_ vs. t_2_: 5.38 vs. 5.65; t_3_ vs. t_4_: 5.38 vs. 5.61; *P* < 0.05), but it was higher than that at T_0_ or T_3_ (T_0_ vs. t_2_: 5.26 vs. 5.65; T_0_ vs. t_4_: 5.26 vs. 5.61; T_3_ vs. t_2_: 5.34 vs. 5.65; T_3_ vs. t_4_: 5.34 vs. 5.61; *P* < 0.05).

**Table 4:** Ultrasound measurement of ONSD

	T_0_	T_1_	t_0_	t_1_	t_2_	t_3_	t_4_	T_3_	*P*
Group A (*n* = 25)	5.32 ± 0.36	5.37 ± 0.32	NA	NA	NA	NA	NA	5.32 ± 0.30	0.864
Group B (*n* = 28)	5.33 ± 0.32	5.37 ± 0.30	5.44 ± 0.31	NA	NA	NA	NA	5.38 ± 0.29	0.527
Group C (*n* = 28)	5.26 ± 0.28	5.32 ± 0.30	5.37 ± 0.31	5.38 ± 0.29	5.65 ± 0.28^abcd^	5.38 ± 0.30	5.61 ± 0.28^abcd^	5.34 ± 0.29	< 0.001

Data are presented as the mean ± standard deviation (mm)

*NA* not applicable; *ONSD* optic nerve sheath diameter

^a^*P*<0.05, ^b^*P*<0.05, ^c^*P*<0.05, ^d^*P*<0.05 compared to T_0_, T_3_, t_1_, t_3_, respectively

Table 5 shows the serum levels of GFAP and UCHL1 in the three groups at different time points. Both of them increased significantly in each group with prolonged operative time (*P* < 0.001, each). At T_1_, there were no significant differences in GFAP or UCHL1 levels among the three groups. At T_2_, the mean serum level of GFAP in Group B was the lowest (399.16 vs. 360.93 vs. 389.12 pg/mL, *P* = 0.042), but a significant difference was only detected between Group A and B. The mean serum level of UCHL1 in Group B was significantly lower than that in the other two groups (828.16 vs. 661.96 vs. 782.00 pg/mL, *P* = 0.001), which was comparable between Group A and C. Similarly, at T_3_, the mean serum level of GFAP was significantly lower in Group B than in the other groups (459.24 vs. 396.68 vs. 431.96 pg/mL, *P* = 0.002), which was comparable in the latter two groups. The mean serum level of UCHL1 was the highest in Group A and the lowest in Group B (1223.00 vs. 849.21 vs. 1068.50 pg/mL, *P* < 0.001).

**Table 5:** Intraoperative serum levels of GFAP and UCHL1

		Group A (*n* = 25)	Group B (*n* = 28)	Group C (*n* = 26)	*P*
GFAP	T_1_	328.68 ± 54.50	325.79 ± 55.82	336.58 ± 61.04	0.776
	T_2_	399.16 ± 55.40^*^	360.93 ± 56.71^a*^	389.12 ± 57.32^*^	0.042
	T_3_	459.24 ± 56.37^*#^	396.68 ± 55.78^a*#^	431.96 ± 71.44^b*#^	0.002
UCHL1	T_1_	422.60 ± 165.27	413.43 ± 172.77	434.58 ± 186.98	0.906
	T_2_	828.16 ± 134.20^*^	661.96 ± 166.73^a*^	782.00 ± 177.36^b*^	0.001
	T_3_	1223.00 ± 126.37^*#^	849.21 ± 175.51^a*#^	1068.50 ± 167.71^ab*#^	< 0.001

Data are presented as the mean ± standard deviation (pg/mL)

*GFAP* glial fibrillary acidic protein; *UCHL1* ubiquitin carboxyl-terminal hydrolase isozyme L1

^a^*P*<0.05 compared to Group A, ^b^*P*<0.05 compared to Group B at the same point in time

^*^P < 0.05, ^#^*P* < 0.05 compared to T_1_ and T_2_ within each group

## Discussion

Our study investigated the effects of intraoperative LPV on respiratory function and the incidences of postoperative complications in emergency TBI patients. The results demonstrated that continuous intraoperative administration of small VT + PEEP could improve oxygenation and respiratory mechanics parameters, decrease the incidence of PPCs, and lower the increase in posttraumatic serum levels of brain injury markers. However, implementing intermittent RMs might disturb intraoperative cerebral hemodynamics, leading to fluctuations in ICP.

Small VT ventilation (6–8 mL/kg CBW) now serves as the respiratory care standard for ARDS patients in the ICU. A consensus has been formed that it is also suitable for patients with healthy lungs in the operating room [12, 13]. An animal experiment showed that small VT ventilation could more effectively promote the oxygenation of rats with brain injury than large VT ventilation [14]. Furthermore, large VT ventilation in TBI patients might be associated with the occurrence of ARDS, and its incidence increased to some extent with VT [15]. However, a single use of small VT causes periodic alveolar collapse of local lung tissues, thus increasing the risk of atelectasis. Interestingly, this adverse effect can be offset by combining PEEP and/or RMs, which would inevitably involve positive pressure ventilation and might adversely affect ICP and CPP [16–18]. However, another study showed that high PEEP (5–15 cmH_2_O) could improve PaO_2_ of local brain tissue without affecting ICP and CPP in patients with TBI and ARDS [19]. In summary, the interaction between the lung and the brain poses an important challenge to ventilation management in TBI patients. The optimal ventilation strategy for TBI patients requires in-depth discussion.

In this study, two groups of TBI patients were treated with LPV: small VT (8 mL/kg CBW), intraoperative continuous administration of 5 cmH_2_O PEEP. Some were given RMs before opening and after closing the endocranium. Our results showed that intraoperative application of small VT + PEEP or small VT + PEEP + RMs improved oxygenation and pulmonary compliance in TBI patients compared to those treated with conventional MV. However, no significant differences in PaO_2_ and Cdyn were found between two intervention groups, suggesting that RMs might not provide further improvement on the basis of PEEP. Ppeak, close to airway pressure, reflects the dynamic compliance of respiratory system. Recently, Pplat was recommended as a better predictor of barotrauma and VALI, as it is closer to alveolar pressure and reflects the static compliance of the respiratory system [20]. Our results showed that patients who intervened with LPV had lower Ppeak and Pplat than those receiving conventional MV, suggesting that the implementation of LPV improved respiratory mechanics parameters and contributed to relieving barotrauma and PPCs. However, there was a rise in PaCO_2_ at the end of surgery in patients receiving LPV, which may be explained by CO_2_ retention resulted from reduced periodic alveolar collapse and expansion following small VT. However, the small rise could be considered a compensatory state that would not cause obvious pathological damage [21]. The incidence of intraoperative hypoxemia was lower in the two intervention groups, while cardiovascular adverse reactions were more frequently observed in the small VT + PEEP + RMs group. Since intrathoracic pressure increased rapidly in a short time, RMs-treated patients generated a decrease in blood volume returning to the heart and cardiac output [16]. Collectively, although small VT + PEEP + RMs can improve intraoperative oxygenation and respiratory mechanics parameters in TBI patients, RMs may cause adverse effects on hemodynamics, while small VT only combined with PEEP can improve lung function without affecting their circulatory stability.

PPCs are the most common mid-term complications after major surgery and strongly linked to clinical prognosis [22]. An observational study in 29 countries named LAS VEGAS showed that 80 % severe TBI patients developed PPCs [7]. Serious pulmonary complications, like ARDS, NPE or VALI, are associated with high mortality, unfavorable neurological outcomes, longer ICU retention and longer hospital stays in TBI patients [23]. We investigated the incidence of pulmonary complications within 30 days postoperatively. The incidences of total PPCs, hypoxemia, pulmonary infection, and atelectasis in LPV-intervened patients were significantly lower than those in patients receiving conventional MV, which was consistent with the Marret E et al. [24] study. Moreover, using LPV significantly reduced postoperative ventilation time, which might be attributed to the improvement of intraoperative respiratory function and decreased risk of PPCs by small VT combined with PEEP and RMs. The GOSE score is usually used to evaluate the degree of disability and neurological prognosis of TBI patients [25]. Our results showed no significant differences in GOSE scores among the three groups after 30 days, probably owing to a short follow-up time and limited sample size. Postoperative neurological complications and hospital stays were comparable among the three groups, possibly because improving the ventilation strategy alone hardly achieved a breakthrough in the neurological outcomes of TBI patients. Surgical factors, the quality of nursing, and family economical state all need to be considered comprehensively.

The greatest concern about TBI patients with respect to perioperative LPV is that it may have an adverse effect on ICP and CPP. Ultrasound measurement of ONSD is a novel noninvasive method that is widely used to dynamically and rapidly assess changes in ICP. It has a close correlation with canonical direct intubation in the ventricle to estimate intracranial hypertension [26, 27]. Our ultrasound results showed no significant changes in ONSD at any time in either conventional MV or small VT + PEEP group, suggesting that intraoperative continuous administration of 5 cmH_2_O PEEP did not affect patients’ ICP. Mascia et al. [15] randomly applied 5 cmH_2_O or 10 cmH_2_O PEEP in 12 patients along with brain injury and ARDS. They found that the PEEP level, which was insufficient to give rise to excessive alveolar expansion or an evident increase in PaCO_2_, had no significant impact on ICP and could safely improve oxygenation. Likewise, if the PEEP value was lower than ICP during MV, the elevation of intrathoracic pressure within a certain range would not increase ICP [28]. Therefore, 5 cmH_2_O PEEP was applied in our study, not visibly increasing ICP, because it did not cause alveolar hyperinflation or it was less than patients’ ICP. However, RMs could rapidly expand the alveoli in a short period and increase the pressure in the thoracic cavity, which blocked the return of systemic circulation to the right atrium (cerebral venous reflux) and eventually increased ICP. After ceasing RMs, the intrathoracic pressure dropped to normal, and the patient’s ICP decreased accordingly [29]. Consistent with previous findings [30], a single RM transiently increased ONSD, which returned to the baseline within 5–10 min, indicating that RMs had the risk of elevating ICP in TBI patients.

Immediately after acute brain injury, astrocytes undergo mechanical deformation or local necrosis, leading to an increased serum concentration of GFAP [31–33]. UCHL1 is a neuron-specific cytosolic enzyme, and its serum level in acute phase of cerebral injury is strongly correlated with the severity of damage [34]. Combined testing of GFAP and UCHL1 could more accurately diagnose the severity of brain damage and predict the long-term prognosis [35]. Here, postoperative serum levels of GFAP and UCHL1 in each group were higher than before. This may be because they were released from necrotic cells and accumulated with the posttraumatic time course [36]. Furthermore, both serum levels of GFAP and UCHL1 in Group B were lower than those in the other groups at T_3_. PEEP can improve oxygenation and reduce the release of inflammatory mediators from the lung and brain, thereby alleviating secondary injuries. Interestingly, additional RMs posed a large continuous positive airway pressure in a short time, which had an adverse effect on cerebral hemodynamics that neutralized favorable results of implementing PEEP. Hence, intraoperative application of small VT + PEEP could prevent further brain damage in TBI patients to some extent. Whether RMs played the same role remained further exploration.

Several limitations in our study should be noted. First and foremost, this study reported the results of a health care intervention on human participants, which should be registered before enrollment of the first participant. We must acknowledge our negligence of the prospective registration, and made a retrospective registration to complete our unfulfilled registration obligations and responsibilities. Second, actual values and accurate changes in ICP were unable to be obtained from ultrasound measurement of ONSD. Third, our results may not be applicable to other neurosurgical patients, such as those with intracranial tumors, craniocerebral injury in the sitting position during surgery, spontaneous cerebral hemorrhage, etc. Fourth, during the study period, especially before the operation, some severe patients were treated with mannitol and dexamethasone due to their conditions, which might consequently affect the results. Fifth, the 30-day postoperative follow-up was unable to accurately evaluate the long-term survival and quality of life of TBI patients. Last, it was a single-center study with a limited sample size. Large-scale clinical trials are needed in the future to validate the impact of intraoperative LPV on TBI patients.

## Conclusions

Intraoperative continuous administration of small VT + PEEP is beneficial to TBI patients, manifesting as improved oxygenation and respiratory mechanics parameters, decreased incidences of PPCs, and smaller increases in posttraumatic serum levels of brain injury markers. However, additional RMs should be cautiously applied in these patients, since they are prone to disturbing intraoperative cerebral hemodynamics.

## Data Availability

The datasets used and/or analyzed during the current study are available from the corresponding author on reasonable request.

## Abbreviations

TBI: Traumatic brain injury
NPE: Neurogenic pulmonary edema
VALI: Ventilator-associated lung injury
VT: Tidal volume
CBW: Corrected body weight
LPV: Lung-protective ventilation
PEEP: Positive end-expiratory pressure
Pplat: Airway plateau pressure
ARDS: Acute respiratory distress syndrome
ICU: Intensive care unit
MV: Mechanical ventilation
PPCs: Postoperative pulmonary complications
RMs: Recruitment maneuvers
ICP: Intracranial pressure
CPP: Cerebral perfusion pressure
BMI: Body mass index
ASA: American Society of Anesthesiologists
P_ET_CO_2_: End-tidal carbon dioxide partial pressure
SpO_2_: Oxygen saturation
PaO_2_: Arterial partial pressure of oxygen
PaCO_2_: Arterial partial pressure of carbon dioxide
Cdyn: Pulmonary dynamic compliance
Ppeak: Airway peak pressure
MAP: Mean arterial pressure
SBP: Systolic blood pressure
GFAP: Glial fibrillary acidic protein
UCHL1: Ubiquitin carboxyl-terminal hydrolase isozyme L1
ONSD: Optic nerve sheath diameter
GOSE: Glasgow Outcome Scale Extended
